# Acknowledgment to Reviewers of *Antibiotics* in 2020

**DOI:** 10.3390/antibiotics10020111

**Published:** 2021-01-25

**Authors:** Antibiotics Editorial Office

**Affiliations:** MDPI, St. Alban-Anlage 66, 4052 Basel, Switzerland

Peer review is the driving force of journal development, and reviewers are gatekeepers who ensure that *Antibiotics* maintains its standards for the high quality of its published papers. Thanks to the cooperation of our reviewers, in 2020, the median time to first decision was 13.6 days and the median time to publication was 33 days. The editors would like to express their sincere gratitude to the following reviewers for their precious time and dedication, regardless of whether the papers were finally published:
Abbina, SrinivasLiebman, MichaelAbd El-Aziz, Tarek MohamedLin, HaishuAbdelmohsen, Usama RamadanLin, Hung-YinAbdul-Aziz, HafizLin, WeifengAbia, Akebe Luther KingLin, Ying-ChiAbraham, Wolf-RainerLin, Yi-TsungAbram, MajaLin, Yusen EasonAbuillan, WasimLinciano, PasqualeAbu-Niaaj, LubnaLionetti, VincenzoAdamczyk-Popławska, MonikaLipke, PeterAdamczyk-Woźniak, AgnieszkaLipman, JeffreyAdewole, DeborahLipner, Ettie M.Adhikari, RajanLiu, Chung-JungAdil, MohdLiu, JinxinAdriaenssens, EvelienLiu, LiangliangAfonso, FernandoLiu, XiaoqiangAfrashtehfar, KelvinLiu, XiaoxiAgnieszka, SzopaLiu, XiaoyunAgüero-Chapin, GuillerminLiu, YangAguilar-Caballos, María PazLiu, YanhongAhern, HollyLiu, YixiangAhn, JuheeLiu, ZhipingAimanianda, VishukumarLlama-Palacios, AranchaAkabayov, BarakLo Giudice, GiuseppeAkbar, SaminaLøbner-Olesen, AndersAlafiatayo, RuthLodise, ThomasAlam, Md KausarLollobrigida, MarcoAlam, Mohammad AbrarLoncaric, IgorAlao, John PatrickLondono, BerlinAlbericio, FernandoLopatkin, Allison J.Aldred, KatieLopez Seijas, JacoboAlduina, RosaLópez, Kelly Johana FigueroaAlex, AnoopLopez-Jornet, PiaAlfandari, SergeŁopusiewicz, ŁukaszÄlga, AndreasLordan, RonanAl-Hasan, Majdi N.Lorenzo-Gómez, Maria-FernandaAlkan, MichaelLoretz, BrigittaAllegaert, KarelLounsbury, NicoleAlmeida, Luis EduardoLous, JørgenAlsager, Omar A.LOZNIEWSKI, AlainAlshabib, EbtihalLu, ShaoyongAlves, DianaLubian, Alicia FajardoAlves, ElianaLuce-Fedrow, AlisonAmarelli, CristianoLuciana, RossiAmi, DilettaLudwiczak, AgnieszkaAnan, GoLunge, Vagner RicardoAnastasio, AnielloLupi, Saturnino MarcoAncuceanu, RobertLupien, AndréanneAnderegg, UlfLutter, ErikaAndrade, José CarlosLütticken, RudolfAndreev, KonstantinM Ghazi, IslamAndrzej, NowickiMa, ChengbangAngelici, Maria CristinaMacori, GuerrinoAngelillo, Italo FrancescoMaddi, AbhiramAngelini, PaolaMaggi, FilippoAngelova, SilviaMagiorkinis, EmmanouilAngiolella, LetiziaMagnusson, UlfAntolak, HubertMahan, MichaelAntoñanzas, FernandoMahato, NeelimaAntunes, JoanaMainar-Jaime, Raúl C.Apostolakos, IliasMaisetta, GiuseppantonioArana, InesMajewska, AnnaArancibia, MirariMajewski, SebastianAraniciu, CătălinMajkowska-Skrobek, GrażynaArastehfar, AmirMajtan, JurajArkadiusz, DziedzicMajtner, TomášArmalyte, JulijaMakarewicz, WojciechArmengot-Carceller, MiguelMakvandi, PooyanArndt, Patrick G.Malagon, FranciscoArnscheidt, JoergMaldonado, JuanArslan, MuhammadMalik, RavinderArtini, MarcoMallipeddi, SrikrishnanArtyukhin, AlexMalm, AnnaAsahara, HaruyasuMalwal, Satish R.Asai, TetsuoMamouni, KenzaAsano, NaokiMan, AdrianAshour, HossamManca, Maria LetiziaAskes, SvenManchester, AlisonAspatwar, AshokManchia, MirkoAthanassopoulos, ConstantinosMancianti, FrancescaAtobe, MasakazuMancuso, MoniqueAttili, Anna RitaMangiaterra, GianmarcoAugustine, Swinburne A. J.Manna, SaikatAugustyniak, AdrianManolescu, Loredana Sabina CorneliaAung, Meiji SoeMantadakis, ElpisAvrani, SaritMantzoukas, SpiridonAwad, MilenaMaraolo, Alberto EnricoAyoade, FolusakinMarc, GabrielAzam, Aa HaerumanMarć, Małgorzata AnnaAzzimonti, BarbaraMaresca, MarcBabii, OlegMari, MicheleBabulak, EduardMárialigeti, KárolyBach, HoracioMarin, EliaBąchor, RemigiuszMarkovska, RumyanaBachy, Christine MMarkowicz-Piasecka, MagdalenaBae, TaeokMarques, ClaudiaBagattini, MariaMarsot, AmélieBai, RenrenMartínez, José LuisBaig, Mohammad HassanMartini, CeciliaBaindara, PiyushMartino, Piera AnnaBajusz, DávidMarzuillo, CarolinaBaker, Jonathon L.Mascellino, Maria TeresaBakhit, MinaMasci, Valentina LaghezzaBaldin, ClaraMascitti, MarcoBaldy-Chudzik, KatarzynaMashruwala, AmeyaBalhaddad, Abdulrahman A.Massella, DanieleBallini, AndreaMateos, JesúsBanfi, GiuseppeMathur, HarshBang, Jeong KyuMatkowski, AdamBankoti, KamakshiMatowicka-Karna, JoannaBanks, LoriMatta, Murali KrishnaBanskota, Arjun H.Mattila, SariBarata, PedroMauritzen, Jesper JuelBarberis, AntonioMayr, JohannesBarbosa, Raquel De MeloMazumder, KishorBarchitta, MartinaMazur, MarcelinaBarciszewska, Anna-MariaMazzone, GloriaBardají, EduardMcEvoy, JamesBar-Joseph, MosheMcFeeters, RobertBarlow, JohnMcGee, Edo-abasi U.Barmparis, Georgios D.McIntosh, E. David G.Barnard, Ross T.McLean, RobertBarrasso, RobertaMcMaughan, DarcyBartosik, Aneta AgnieszkaMcVicker, GarethBasavegowda, NagarajMeesters, KevinBassolé, Imaël Henri NestorMenanteau-Ledouble, SimonBatoni, GiovannaMendes, RafaelBattiato, AlfioMenzel, LorenzoBatzias, GeorgiosMercier, CorinneBaumann, Jean-SébastienMertas, AnnaBaumgarten, ThomasMesa, FranciscoBeaurepaire, AlexisMeshram, ChetanBednarczyk-Cwynar, BarbaraMesquita, JoãoBednárová, LucieMessi, PatriziaBednarski, Patrick J.Mestre, Ana S.Behzadi, PayamMeštrović, TomislavBeier, Ross C.Metzinger, LaurentBelguesmia, YanathMeyssonnier, VaninaBelitsky, Jason M.Mézes, MiklósBelyi, YuryMidwinter, AnneBenarba, BachirMiguel, Trinidad DeBender, KathrynMihai, MaraBenedec, DanielaMilanova, AneliyaBenedetti, FrancescaMilewski, SławomirBenkő, RiaMinami, MasaakiBennett, NoleenMinnelli, CristinaBenton, Angela H.Miotla, PawelBernal, Freddy AlexanderMirijello, AntonioBernardi, SaraMishra, MeerambikaBernardini, GiuliaMitchell, MiguelBertelloni, FabrizioMitsea, AnastasiaBerti, Andrew DavidMiyamoto, HirotakaBertoglio, FedericoMiyanaga, AkimasaBerzins, AivarsMiyazaki, MotoyasuBessa, LucindaMizuno, Cassia SBessho, KazuhisaMlaga, Kodjovi D.Bettini, SimonaMoawad, Amira A.Bevilacqua, AntonioMoffa, Matthew A.Bezirtzoglou, EugeniaMofidfar, MohammadBEZU, LucilliaMognetti, BarbaraBhat, KruttikaMohammed, ManalBhattacharjee, MrinalMohanram, HariniBhattacharya, SomanonMohd Hanafiah, KhayriyyahBi, YangMojsoska, BiljanaBialonska, DobroslawaMöller, SörenBianco, ArmandodorianoMoloney, Mark G.Bielas, RafałMombelli, Andrea W.Bielenica, AnnaMomin, Mohammad Abdul MotalibBierbaum, G.Monaghan, ThomasBiernasiuk, AnnaMoncalián, GabrielBiezen, RubyMonteiro, CláudiaBikov, AndrasMontiel, José MaríaBilal, MuhammadMorales Suárez-Varela, María M.Billamboz, MurielMoran-Gilad, JacobBirsa, Mihail LucianMoreira, CristianaBlatzer, MichaelMorel, Chantal M.Błażewicz, AnnaMorgenstern, ChristianBloch, SylwiaMorimoto, KinjiroBloemberg, GuidoMorozova, Vera V.Bloise, NoraMorris, Andrew ConwayBochniarz, MariolaMorrissey, HanaBocian, AleksandraMorroni, GianlucaBoffano, PaoloMorse, DanielBogomolnaya, Lydia M.Mot, AugustinBøgwald, JarlMourad, AhmadBohn, ErwinMourão, JoanaBojinov, BojinMozos, IoanaBok, EwaMuccilli, VeraBondoc, IonelMukherjee, DwaipayanBondon, ArnaudMukherjee, RatnadeepBonelli, FrancescaMulinacci, NadiaBongiorno, DafneMuller, CécileBonifacio, Maria AddolorataMunang’andu, Hetron MweembaBookstaver, P. BrandonMunteanu, Florentina-DanielaBoomer, Jonathan S.Murakami, TeruoBorbas, AnikoMurias, MarekBoroń-Kaczmarska, AnnaMurri, RitaBorrelli, LucaMusso, IoanaBortoluzzi, CristianoMuthu Karuppan, Mohan KumarBorza, TudorMutreja, IshaBosch, ChristineMyung, HeejoonBostik, PavelNaber, KurtBotelho, JoãoNagy, MiklósBottomley, AmyNair, Meera SurendranBoucher, DaveNanjappa, Som G.Bouquillon, SandrineNante, NicolaBourgeois, DenisNarra, Hema PrasadBovera, FulviaNavas, JesúsBowden, BruceNazzaro, FilomenaBozzo, GiancarloNeelakantan, PrasannaBracher, FranzNemeș, Roxana MariaBraczkowski, RyszardNeo, Ana G.Brandt, CurtisNewman, DJBraun, SergeNg, Kenneth Kai-SingBraun, VolkmarNguyen, TrieuBright, RichardNicosia, AldoBrillowska-Dąbrowska, AnnaNie, ChuanxiongBrischke, ChristianNiedźwiedzka-Rystwej, PaulinaBroecker, FelixNielsen, Peter EigilBroens, EMNigro, ErsiliaBrogi, SimoneNikolaivits, EfstratiosBrown, Bryan R.Nikolaou, ElissavetBrown, GeoffreyNikolova, MariaBrown, TeaganNimse, Satish BalasahebBrukner, IvanNing, XuhuiBrunel, Jean MichelNiu, DongyanBrycki, BogumilNiyonsaba, Dr. FrancoisBucekova, MarcelaNoh, KeumhanBuckner, MichelleNohe, AnjaBudachetri, KhemrajNomoto, RyoheiBugarin, AlejandroNørskov-Lauritsen, NielsBuil, Jochem B.Nostro, AntoniaBujňáková, DobroslavaNovick, Richard P.Bukowski, MichalNovo-Veleiro, IgnacioBulet, PhilippeNový, PavelBull, JimNowacka, MalgorzataBungau, SimonaNowacka, MariaBuommino, ElisabettaNowak, AdrianaBurdukiewicz, MichałNowakiewicz, AnetaBurger, Michael CNowicka, GrażynaBurugupalli, SatvikaNtalli, Nikoletta G.Bushell, MichaelNüesch-Inderbinen, MagdalenaBustos-Martínez, JaimeNyerges, GyorgyiButaye, PatrickObreza, AlešButler, DavidObuobi, Sybil Akua OkyerewaButtimer, ColinO'Connor, MichaelBuyle, FrankyOdoch, TerenceC. J. M. D. S. Menezes, JoséOe, MakatoCacciotti, IlariaOechslin, FrankCádiz-Gurrea, María De La LuzOelschlaeger, PeterCaeiro, Maria FilomenaOgawa, AkikoCafarchia, ClaudiaOgórek, RafałCaffrey, PatrickOgrodowczyk, AnnaCai, WenlongOh, JaeseongCaimano, Melissa J.Ohta, NaomiCajal, YolandaOkada, YoshiharuCalcutt, MichaelOkafor, FlorenceCaldara, MarinaOkamoto, MasamiCalendar, RichardOkorski, AdamCalina, DanielaOkunade, Adewole L.Callanan, Michael J.Olejníková, PetraCama, JehangirOligbu, GodwinCameron, David RobertOliveira, David DeCampana, RaffaellaOlson, LinusCampos, Débora A.Omar, Ahmad A.Campos, Maria Jorge GeraldesOng, Cheryl-lynnCaneiras, CátiaOnnis, ValentinaCapasso, RaffaeleOnyeaka, HelenCapparè, PaoloOppliger, AnneCarabetta, Valerie J.Orkusz, AgnieszkaCardoso, SusanaOrlando, GabriellaCarlos, Ana RitaOrtega, Joseph ThomasCarmeli, YehudaOstos, Francisco JoséCarpes, Solange TeresinhaOves-Costales, DanielCarreras, JoaquimØvrehus, Marius ACarrilho, EuniceOzaki, HiroichiCarullo, GabrieleOzarowski, MarcinCaruso, GerardoP. Sagona, AntoniaCasanova, Alfredo GinésPacyga, KatarzynaCasertano, MarcelloPaczkowski, JonCastaldi, SilvanaPadnya, PavelCastle, AlanPagliarani, AlessandraCastro, JoanaPagniez, FabriceCattoir, VincentPailhoriès, HélèneCaulfield, MalcolmPais, PedroCavoretto, PaoloPaiva, SandraCelenza, GiuseppePaixão, PauloCerbo, Alessandro DiPajunen, MariaCeroni, DimitriPal, ChandanChakraborty, BrintaPallecchi, LuciaChamakura, Karthik R.Palma, Paulo JorgeChan, Ben C. L.Panaro, Maria AntoniettaChandrasekaran, MurugesanPancholi, VijayChao, Chia-TerPandit, SantoshChao, Chih-HuaPanzella, LuciaChapeland-Leclerc, FlorencePapadimitriou Olivgeris, MatthaiosCharani, EsmitaPapagiannitsis, Costas C.Charoensutthivarakul, SitthivutPapaioannou, EmmanouilCharvat, RobertPaparella, Ashleigh S.Chatterjee, SatabdiPapoutsis, KonstantinosChaudhri, VirendraPapp, JohnChen, CharlesPapp-Wallace, Krisztina M.Chen, HaoqingParamythiotou, ElizabethChen, LiliPardo, FatimaChen, Pin-jungParikesit, Arli AdityaChen, Po-WenPark, Jun-BeomChen, WangxuePark, Ki HwanChen, XuPark, Se ChangChen, ZhaoPark, Seung-ChunCheng, Jya-WeiParlapani, Foteini F.Cheng, Vincent Y-HParrish, RichardCheng, Yuan-BinParthasarathy, AnutthamanCheng, ZishuoPassalacqua, KarlaCheshenko, NataliaPatatanian, EdnaChevrot, RomainPatching, Simon GChiarelli, LaurentPatel, HardikkumarChien, Shih-ChangPaterson, Gavin K.Chifiriuc, CarmenPathak, AshishChikh Ali, MohamadPatil, VeerupaxagoudaChilds-Kean, LindseyPatini, RomeoCho, HanjinPatterson, JenniferChochlakis, DimosthenisPatyra, EwelinaChoi, JungilPavasini, RitaChoi, Ki ChoonPavoni, MatteoChoi, MyeongjinPawar, Shrikant D.Choi, Youkyung H.Pawlik, AndrzejChow, Franklin Wang-NgaiPeckham, Gabriel D.Chowdhury, Imran HussainPecyna, PaulinaChowdhury, NityanandaPedatella, SilvanaChristodoulides, MyronPellegrini, MarikaChristoforides, EliasPeng, ChaoChrysanthakopoulos, Nikolaos A.Pennone, VincenzoChurchward, Colin P.Perdigão, JoãoChwalibog, AndréPereira, CarlaChwialkowska, KarolinaPérez Prieto, DanielCicciù, MarcoPérez-Cobas, Ana ElenaCieslar, GrzegorzPérez-Gracia, María-TeresaCilas, ChristianPérez-Martínez, ClaudiaCirocchi, RobertoPerinelli, Diego RomanoCitores, LuciaPero, RaffaelaČižman, MilanPerucka, IrenaCłapa, TomaszPeruzy, Maria FrancescaClarke, DavidPesce, PaoloClement, CristinaPetraitis, VidmantasClemente, LurdesPhee, LynetteColeine, ClaudiaPiano, MartinaColliers, AnneliesPichon, MaximeCollins, DeirdrePiepenbrink, KurtConcilio, SimonaPietrella, DonatellaConnell, Sean R.Piluzza, GiannellaConrads, GeorgPinela, JoséConstantinos, PapagiannitsisPinheiro, JorgeContaldo, MariaPinho, EvaContardi, MarcoPinto, Diana CláudiaCopolovici, Dana MariaPinto, LorisCornelison, Christopher T.Pinto, SandraCornelissen, Cynthia NauPinzaru, IuliaCorral, PaulinaPiras, FrancescaCorrò, MichelaPires, AldaCorvo, Marta C.P.C.Pires, JoãoCorzo-León, Dora E.Pisanu, ClaudiaCosma, PinalysaPisapia, PasqualeCosmin Mihai, VesaPisk, JanaCosta, Ana RitaPitarch, AidaCostantini, LaraPiu, SahaCottreau, Jessica M.Placido, AntonioCouto, NatachaPlano, Laura DeCoyne, LucyPlatt, ThomasCozza, GiorgioPletzer, DanielCrnkovic, AnaPlonka, PrzemyslawCromwell, DavidPlotka, MagdalenaCrossman, LisaPlotniece, AivaCuadra, GiancarloPobiega, KatarzynaCui, QingbinPoddighe, DimitriCui, ZhengPogorelić, ZenonCuny, Gregory D.Pokorn, MarkoD’Accolti, MariaPolidori, PaoloD’Agostino, DonatoPolyak, StevenD’Amico, RamonaPoma, AdolfoD’Elios, Mario MilcoPomari, ElenaDa Silva, Gabriela JorgePopa, Mircea IoanDabrowski, JanuszPopov, AntonDahiya, SatinderPorcelli, FernandoDahlén, GunnarPospíšilová, ŠárkaDai, JiangkunPostma, MarteenD'alfonso, LauraPotaniec, BartłomiejD'Andrea, Marco MariaPotron, AnaisDanielson, NeilPotter, BethDante, SilviaPozdin, VladimirDanziger, Larry HPozzi, CeciliaD'Argenio, ValeriaPrakasarao Kovvasu, SuryaDash, Ranjeet PrasadPresentato, AlessandroDaubert, Diane M.Prigigallo, Maria IsabellaDaugelavicius, RimantasPrincipe, LuigiDavid, MichaelProft, ThomasDavideau, Jean-LucProkesch, Bonnie C.Davies, Bryan WilliamProkhorov, NikolaiDay, Andrew S.Prosen, HelenaDe Biasi, Margherita G.Pruess, Birgit M.De Boni, Annalisa De BoniPrunty, Leesa M.De Briyne, NancyPurcell, Erin B.De Jonghe, StevenPuri, SumantDe La Herrán, RobertoPurkrtová, SabinaDe La Lastra, José Manuel PérezQi, YanfeiDe Lagarde, MaudQiao, MingyuDe Loof, HansQin, HuaDe Masi, LuigiQuinto, Emiliano JoséDe Mendonça, Dina I. M. D.Rabanal Anglada, FrancescDe Re, VallìRadaram, BhaskerDe Sire, AlessandroRadko, LidiaDe Vita, DanielaRadlinska, MonikaDe Vito, CorradoRadoja, IvanDe Zotti, MartaRadu, FierascuDea-Ayuela, MaríaRaffa, DemetrioDea-Ayuela, María AuxiliadoraRagonese, MauroDean, FrankRagusa, AndreaDeb, SubrataRahimi, ParvanehDec, MartaRahman, AzizurDella Sala, GerardoRahman, M. AzizurDell'olmo, ElianaRai, NavneetDelorme, VincentRailean-Plugaru, VioricaDeme, PragneyaRaizman, RoseDemetrescu, IoanaRajendran, Jagathesh ChandraDemopoulos, Vassilis J.Ramakumar, KinthadaDenina, MarcoRamamoorthy, AyyalusamyDering Anderson, AllyRamasamy, MohankandhasamyDeshpande, GauraviRamezanpour, MahnazDeslouches, BerthonyRamírez, Ana SofíaDestache, Christopher J.Ramos-Vivas, JoséDhakal, SantoshRampacci, ElisaDhariwala, MiqdadRanzato, EliaDi Giallonardo, FrancescaRaposio, EdoardoDi Maro, AntimoRatajczak, MagdalenaDi Onofrio, ValeriaRather, IrfanDi Pietro, MarissaRatiu, AndreeaDi Salle, AnnaRatusz, KatarzynaDiana, PatriziaRaudabaugh, DanielDianez Martinez, FernandoRautiainen, LindaDíaz Martínez, MargaritaRavaioli, StefanoDiaz-Marrero, Ana R.Raval, YashDickman, RachaelRavindran, VelmuruguDíez, DavidRayson, Gary D.Díez-Aguilar, MaríaRazvan-Cosmin, PetcaDijkstra, BaukeRechkoblit, OlgaDillard, Joseph P.Redfern, LaurenDimitriou, GabrielRedrejo Rodriguez, ModestoDinache, AndraRegal, PatriciaDingley, Andrew J.Rego, RyanDinos, GeorgiosRehman, Md TabishDionisio, GiuseppeRen, YulinDixon, NicholasReva, OlegDixon, Ronald AReverter, EnricDkhar, Hedwin KitdorlangRevolinski, SaraDo Monte, Daniel F. M.Rey, FelisaDobay, OrsolyaReynolds, RobertDocea, Anca OanaReznik, SandraDodds, W. JeanRhodes, Nathaniel J.Doerrler, WilliamRibeiro, Teresa GDomingues, SaraRichter, KatharinaDomínguez Álvarez, EnriqueRijo, PatríciaDonadu, Matthew GavinoRinnerthaler, MarkDong, BoRiska, PaulDong, DongRivero-Müller, AdolfoDong, WeibingRizzi, MenicoDonnarumma, GiovannaRizzo, CarmenDonnelly, Callum GeorgeRobino, PatriziaDonno, DarioRoch, Franz-ferdinandDonta, SamuelRocha, FernandaDorn, ChristophRodríguez Díaz, Juan CarlosDorotkiewicz-Jach, AgataRodríguez, Armando AlexeiDos Santos, Maria Clara VieiraRodríguez-Calleja, Jose MaríaDosoky, NouraRodríguez-Estévez, VicenteDouda, OndřejRodríguez-López, LorenaDoulberis, MichaelRodríguez-Solana, RaquelDrabińska, NataliaRogers, ThomasDrahovská, HanaRogobete, Alexandru FlorinDrlica, KarlRogovskyy, ArtemDruszczynska, MagdalenaRohde, KyleDuarte, AidaRojas, LauraDuca, MariaRojo, Maria AngelesDudek, KatarzynaRomanò, Carlo L.Dudhipala, NarendarRoncada, PaolaDuggan, Brendan M.Roncarati, DavideDukić, WalterRoncevic, TomislavDulf, FranciscRoque, FátimaDuncan, SteveRos, UrisDuong, PhuRosini, ElenaDupureur, Cynthia M.Rostron, AnthonyDurán-Peña, María JesúsRoszczenko-Jasinska, PaulaDuranti, ClaudiaRóżalska, BarbaraDurham, SpencerRóżańska, AnnaĐurović, SašaRubiolo, PatriziaDutta, SanjuctaRuckdeschel, KlausDuval, Raphaël E.Rudnicka, KarolinaDuzgunes, NejatRudra, ArnabDziurkowska, EwelinaRuiz Giardin, Jose ManuelEadie, LauraRuiz Téllez, TrinidadEbadollahi, AsgarRuiz, LeticiaEbert, Timothy ARuiz-Rodríguez, Juan C.Ecke, ThorstenRuntuwene, Lucky RonaldEckroat, ToddRusso, AlessandroEconomou, AnastassiosRusso, DanielaEconomou, VangelisRusso, NinoEedara, Basanth BabuRusso, PasqualeEfremenkova, Olga VRussu, WadeEggers, Christian HRyan, MichaelEichhorn, IngaRybczyńska-Tkaczyk, KamilaEick, SigrunRybtsov, StanislavEkateriniadou, LoukiaRyter, KendalEkiri, Abel B.Sachar, MadhavElena, CriscuoloSadgrove, NicholasElfalleh, WalidSadowska, BeataElkins, KellySagona, SimonaEllett, FelixSaha, Sajal K.Ellis, Terri N.Sahagun, Ana M.El-Seedi, HeshamSahni, Sanjeev K.Elshafie, Hazem S.Sajid, AndaleebElshahawi, SherifSajkiewicz, PawełEnjapoori, Ashwantha KumarSakudo, AkikazuErben, MauricioSaláková, AlenaErmolaeva, SvetlanaSalas, José BlancoErnst, Ulrich RainerSalmeri, MarioEspinosa Padrón, ManuelSalud Bea, Roberto De LaEufemi, MargheritaSalud, SerranoEvangelopoulos, Dr DimitriosSalvatore, SilviaEveillard, MatthieuSambri, AndreaFabiani, RobertoSamuel, EssieFalconi, MaurizioSánchez-Sanchez, ManuelFaleiro, Maria LeonorSander H.J., SmitsFalsini, SaraSandoe, JonathanFan, Ming Z.Sanso, Andrea MarielFan, ZhijinSantaniello, AntonioFang, Shiuh-BinSantarelli, AndreaFantoni, MassimoSantarossa, MaressaFaraone, NicolettaSantos, Ana CláudiaFarone, Mary B.Santos, RicardoFasciana, TeresaSantos, Rita SobralFasina, Folorunso OludayoSantulli, GaetanoFeeney, MorganSaraiva, Raúl G.Felicetti, TommasoSarbini, ShahrulFeliciano, JoanaSarkar, AmritaFeresin, GabrielaSarma, SauravFernandes, LiseteSato, ShuzoFernandez-Martinez, LorenaSato, TakuichiFerran, AudeSaturnino, CarmelaFerrante, ClaudioSaurav, KumarFerrarotti, FrancescoSavage, PaulFerraz, Maria PiaSaviane, AlessioFerraz, RicardoScacco, SalvatoreFerreira, HenriqueSchaaper, RoelFerreira, MarceloSchein, RebeccaFerreira, Paula M.Schierack, PeterFerreira, SusanaSchildberg, Frank A.Ferrer-Espada, RaquelSchildgen, OliverFiedler, Hans-PeterSchito, Anna MariaFierascu, Radu ClaudiuSchlundt, JoergenFierro, José FernandoSchmidt, MartinFiester, SteveSchnabel, UtaFijan, SabinaSchüller, ChristophFilipello, VirginiaSchwan, WilliamFindlay, BrandonSchwarz, LukasFink, BerndSchwarz, StefanFiorillo, LucaSchweigkofler, WolfgangFirth, Clair L.Scipioni, AnitaFisher, Jed F.Scoffone, Viola CamillaFliefel, Riham M.Seca, Ana M. LFlorea, Ana-MariaSecott, TimFlorek, EwaSeidavi, AlirezaFlorindo, Pedro R.Seipke, RyanFloris, RosannaSekara, AgnieszkaFong, KarenSellars, JonathanFonseca, FilomenaSelvaraj, ArokiyarajForsberg, KaitlinSen, KeyaFortunato, LeonzioSendra, EstherForzato, CristinaSeo, JiwonFossey, John S.Serrano, Juan Alfonso AyalaFoster, TimSertić, MirandaFouladkhah, AliyarSerwer, PhilipFragkou, Paraskevi C.Seto, ShigekiFraile, LorenzoSetzer, William N.Franco, AlessiaSevvana, MadhumatiFranco, Carlos M.Sewald, KatherinaFranco, DomenicoShafer, William M.Franco, Marina SantiagoShah, AjitFrank, LuizaShaikh, SibhghatullaFrei, AngeloShakerley, NicoleFrija, LuísShakya, AkhileshFrimodt-Møller, NielsShao, QiuyanFris, MeganSharafutdinov, IrshadFroissart, RémySharkey, LiamFu, PengSharma, AtulFuchs, BethSharma, IshaFuller, AmeliaSharma, ManviFuochi, VirginiaSharma, MeghaFurneri, Pio MariaSharp, Natasha J.Gaglione, RosaShelat, Vishalkumar GGajdács, MárióShelenkov, AndreyGajdosik, Martina SrajerShetty, AmithGallo, MonicaShimomura, HirofumiGálvez Buerba, Eva M.Shin, JongheonGama, SofiaShiota, SeijiGao, ChunxuShirahata, TatsuyaGao, JieShoham, MenachemGaragiola, UmbertoShornick, LaurieGarcia, RonaldShree, SonalGarcía-Cela, EstherShridhar, SurekhaGarcia-Doval, CarmelaShukla, SiddharthGarcia-Gimenez, Maria DoloresSiamidi, AngelikiGarcia-Rubio, RocioSiebert, Hans-ChristianGarcía-Ruiz, AlmudenaSieniawska, ElwiraGarcía-Santos, José AntonioSierra, Josep M.García-Sanz, VerónicaSievers, SusanneGarcía-Suárez, María Del MarSigusch, Bernd W.Gardiner, Laura-JayneSikora, KarolGarner, Bianca L.Sikora, MariuszGarus-Pakowska, AnnaSikorska, EmiliaGatta, LuigiSilva, Marcelo SousaGaudet, AlexandreSilva, Pedro JorgeGautam, UmaSilva, TaniaGavenonis, JasonSilvan, Jose ManuelGeddes-McAlister, JenniferSimecka, Jerry W.Gekenidis, Maria-TheresiaSimeone, PaolaGenilloud, OlgaSimmons, KatieGenova, ChiaraSimonetta, D’ErcoleGenovese, CarloSimonetti, SimonettaGentile, CarlaSimpson, TJGeorgiev, VasilSinger, GeorgGeronikaki, AthinaSingh, ManishGervasi, TeresaSingh, ReemaGestal, MonicaSingh, SukhvinderGestal, Monica CartelleSingh, VineetGharehyakheh, AminSinnott, Catherine J.Ghiciuc, Cristina MihaelaSiodłak, DawidGiacobbe, Daniele RobertoSirobhushanam, SirishaGiammanco, AnnaSistla, SrinivasGiangaspero, MassimoSitarek, PrzemysławGil, RosarioSitarz, RobertGilbride, Kimberley A.Sivakumar, SushamaGillespie, James W.Sivalingam, PeriyasamyGiudice, ValentinaSjöling, ÅsaGiuffrè, MauroSkarzynska, MagdalenaGkentzi, DespoinaSkerlavaj, BarbaraGnanasundram, Sivakumar VadivelSkoglund, ErikGnat, SebastianSkowron, KrzysztofGniewosz, MałgorzataSkwor, TroyGodman, BrianSlavcev, RoderickGoh, ShanŚliżewska, KatarzynaGolemi-Kotra, DasantilaSłomińska-Wojewódzka, MonikaGomes, Ana P.Smith, DavidGomes, Ana T. P. C.Smith, Hayden L.Gomes, Maria SaloméSmith, JessicaGomes, NelsonSmith, LincolnGómez-Barrero, SusanaSmith, Sinead M.Gómez-Salgado, JuanSmułek, WojciechGoněc, TomášSnyder, Orman .LGong, ZifanSoares Silva, Isabel JoaoGonzález-Fandos, María ElenaSobeh, MansourGoodman, Alan G.Sobolewski, JarosławGopegui, Rafael R.Sobotta, ŁukaszGorovits, BorisSocas-Rodríguez, BárbaraGorseta, KristinaSocea, BogdanGosciniak, GrazynaSoengas, Raquel G.Goto, TakaharuSóki, JózsefGotoh, KenjiSolanki, Manoj KumarGotsbacher, MichaelSolano, FranciscoGoytia, MairaSolarino, BiagioGrabarczyk, MałgorzataSolís García Del Pozo, JuliánGrainge, IanSoloski, Mark J.Grande, RossellaSomerville, Greg A.Granica, SebastianSong, JeongminGrassi, AusiliaSong, Yang-HeonGraves, JosephSorensen, JohnGreetham, DarrenSousa, Sílvia A.Gregório, JoãoSouza, PedroGregory, StevenSovadinova, IvaGriffiths, DevinSpadaro, SavinoGrigoryan, LarissaSpanakis, MariosGrimes, Darrell JaySpeck, Peter G.Grimm, W. D.Spiegler, VerenaGrishin, Alexander V.Spires, StevenGrose, Julianne H.Splichal, IgorGrygorcewicz, BartłomiejSroka, ZbigniewGu, ZhenStančiaková, LuciaGuarnieri, GabriellaStarek, MałgorzataGuembe, MariaStasiak-Różańska, LidiaGuerin, FrançoisStein, DanielGugliandolo, ConcettaSteinman, AmirGuminska, JolantaStella, SimoneGunia-Krzyżak, AgnieszkaStepensky, DavidGupta, CharuSter, CélineGupta, KajalSternberg-Lewerin, SusannaGupta, KuldeepStraniero, ValentinaGursoy, MerviStricker, RaphaelGustafson, John E.Stringaro, AnnaritaGutierrez, ClaudeStrohfus, Pamela K.Gutiérrez-Martin, César B.Stromberg, ZacharyHadjikakou, Sotiris KStudzinska-Sroka, ElzbietaHajra, AdrijaStupica, DašaHalperin, JohnSturgill, MarcHamedi, Hamid RezaSturtevant, JoyHammerl, Jens AndreSubramani Paranthaman, BalasubramaniHammoudi Halat, DalalSuckling, Colin J.Han, QianSugawara, AkihiroHan, WenyuSugimoto, MitsushigeHan, ZheSultana Rekha, RokeyaHanania, MichelSungthong, RungrochHanna, Ramy M.Surdacka, AnnaHano, ChristopheSurup, FrankHantke, KlausSuzuki, Jon B.Harada, KazukiSvetlov, MaksimHardy, John GeorgeSvircev, Antonet M.Harnisz, MonikaSweeney, TimothyHarper, David R.Świątek, ŁukaszHarrison, Paul H. M.Świętnicki, WiesławHartke, AxelSyeda, AishaHartman, MariuszSzabó, AndreaHasegawa, MorifumiSzabó, DóraHashimoto, HidekiSzacon, ElzbietaHaslinger, KristinaSzakmany, TamasHassan, Sammer-ulSzczerba-Turek, AnnaHatfull, GrahamSzéll, MártaHearn, Michael J.Szewczyk, KatarzynaHedman, HaydenSzulczyk, DanielHeever, Johan P. Van DenSzultka-Młyńska, MałgorzataHefferon, Kathleen L.Szweda, PiotrHegedűs, CsabaTada, RuiHeinz, WernerTadić, VanjaHek, KarinTagad, HarichandraHelliwell, RichardTahrioui, AliHeney, ClaireTailhades, JulienHeng, Nicholas C.K.Takeda, ShigekiHernalsteens, Jean-PierreTaki, MakotoHernández-León, AlejandroTalukdar, Prabhat K.Hernández-López, HiramTalukder, PoulamiHerzig, VolkerTamaian, RaduHidalgo-Tenorio, CarmenTame, JeremyHiggins, OwenTampa, MirceaHijano, Diego R.Tan, QiHilbert, FriederikeTanaka, KeikoHill-McManus, DanielTang, YulongHindra, HindraTangadanchu, Vijai Kumar ReddyHippensteel, JosephTannous, JoannaHirakawa, HidehikoTao, KaiHo, Chia-cheTao, PanHo, Shih-ChingTao, PengHof, HerbertTarasov, AndreiHofer, Matthias D.Tarazona, FranciscoHoff, RodneyTashima, ToshihikoHoffman, AngelaTavío, María M.Hojjat-Farsangi, MohammadTaylor, DeniseHolbech Jensen, Benjamin AnderschouTaylor, MatthewHolbein, BruceTaylor, NicholasHolien, JessicaTecilla, PaoloHolt, Scott M.Tee, JamesHoltorf, Anke-PeggyTeixeira, PilarHong, Seok HoonTeng, PengHoogland, DominiqueTenhagen, Bernd-AloisHorhat, Florin GeorgeTeoh, LeanneHrast, MartinaTharmalingam, NagendranHrckova, GabrielaThekkiniath, JoseHsiao, Yi-HsuanThiéry, ValérieHsu, Hsin-WeiThies, StephanHu, HelenThikkurissy, SaratHu, JiafenThipe, Velaphi C.Hu, JingjieThirunavukarasu, DeepakHu, JunhuiThomas, WayneHu, XianglongTichaczek-Goska, DorotaHuck, OlivierTinoco, Arthur D.Hudlikar, RasikaTirillini, BrunoHume, AnnaTiwari, Rakesh K.Hung, Shao-WenTkavc, RokHurley, JamesTogawa, AtsushiHurst, Brett L.Toiu, AncaIasur-Kruh, LilachToledo, AlvaroIde, KazukiTolomelli, AlessandraIdnurm, AlexanderTomašič, TihomirIebba, ValerioTomoda, HiroshiIgarashi, YasuhiroTonelli, MicheleIglesias, EmiliaTong, YaojunIijima, NoriakiTongaonkar, Prasad C.Ijiro, KuniharuTonkin-Crine, SarahImai, TomoyaTonolo, FedericaImbery, Terence A.Torrado, Juan J.Imperi, FrancescoTorrent, MarcInchingolo, FrancescoTorres Barcelo, ClaraIoannou, EfstathiaTorres Sangiao, EvaIonita, PetreTorroba, TomasIonut, Ioana A.Tortorella, PaoloIovino, FedericoTorzewska, AgnieszkaIshii, TakahiroTóth, FerencIslam, Md ZohorulToutain, Pierre LouisIslam, Md. TabibulTownsend, EleanorIslam, Salim TimoTrcek, JanjaItenov, Theis SkovsgaardTrček, JanjaIwasaki, TakashiTrincone, AntonioIzawa, Kazuhiro P.Tripathi, AmitIzquierdo-Bueno, InmaculadaTripathi, Jitendra KumarJadeja, RavirajsinhTroutman, Jerry M.Jaehne, Anja KathrinTsioutis, ConstantinosJain, PrashantTsironis, Giorgos P.Jakab, EndreTsugawa, HitoshiJaki, BirgitTsukahara, TakamitsuJakovljevic, MihajloTsur, Avishai MichaelJakubowski, WitoldTudela, JoseJalasvuori, MattiTufarelli, VincenzoJang, SoojinTurner, Raymond J.Janganan, ThamaraiTyson, GregoryJanowicz, AnnaTzeng, Shiang-JongJansen, RolfUchiyama, SatoshiJaskiewicz, MaciejUpadhyay, Kiran K.Jaswal, RajneeshUpdegrove, Taylor B.Jedelská, JarmilaUrban, PhilippeJedziniak, Piotr JanUrbańczyk-Lipkowska, ZofiaJeliazkova, EkaterinaUritu, Cristina MarianaJenssen, HåvardUsai, DonatellaJesudoss Chelladurai, JebaUsher, JaneJi, TianjiaoUsonis, VytautasJiang, Jhih-HangVaclavikova, RadkaJiang, ZiwenVaismoradi, MojtabaJijie, RoxanaVale, Filipa F.Jiménez, CarlosValko, KlaraJiménez-Ortigosa, CristinaValletta, AlessioJing, HuiValm, Alex M.John Molenaar, AdrianVan Aerschot, ArthurJohnson, PaulVan Der Werf, T.S. (Tjip)Join-Lambert, Olivier FVan Le, Hanh ThiJones, George H.Vanderpas, JeanJordao, LuisaVányolós, AttilaJorge, PaulaVarga, CsabaJoseph, SusanVasudevan, AbhinavJoshi, SameerVeeravalli, VijayabhaskarJung, SaschaVeiga, MarianaJung, Tae SungVeiga, PaulaJunquera, LuisVelikova, TsvetelinaJuszczak, LesławVellinga, AkkeK. Karabagias, IoannisVengušt, ModestKačániová, MiroslavaVenkannagari, HarikanthKaczanowski, SzymonVenkatesan, JayachandranKadam, AvinashVennerstrom, JonathanKafarski, PawełVenter, HenriettaKaldhone, PravinVeres, MiklosKalemba, DanutaVersino, ElisabettaKalinowska Lis, UrszulaViapiana, AgnieszkaKalus, KajetanVieyra, Jorge ParedesKamysz, ElżbietaVig, Parminder J. S.Kanamoto, TaiseiVijgenboom, ErikKanematsu, HideyukiVikram, AmitKang, ChanKyuVilar Ares, Maria JKang, SanghoonVilibić-Čavlek, TatjanaKang, WonkuVilková, MáriaKantminienė, KristinaVillari, PaoloKao, Cheng-YenVincent, AntonyKapusta, IreneuszVinuesa, TeresaKarageorgos, SpyridonVirok, Dezső P.Karagouni, Amalia D.Vishe, MaheshKaramonova, LudmilaVitale, MariaKaraś, MagdalenaVlainić, JosipaKarasali, HelenVlasiou, ManosKarbownik, AgnieszkaVouros, IoannisKarcz, DariuszVozza, IoleKarmakar, ParthaWafa, HazemKarnati, Konda ReddyWagle, Basanta RajKarpiński, MirosławWajima, TakeakiKaschina, ElenaWałecka-Zacharska, EwaKashiwagi, AkikoWalker, Robert J.Kasimanickam, Vanmathy R.Walsh, FionaKatada, KazuhiroWan, ThomasKato, HideoWanat, MartaKatsuyama, YoheiWandzik, IlonaKaushik, AshleshaWang, Chao-MinKavaliauskas, PovilasWang, ChengmingKavita, KumariWang, ChihyangKawabata, HideakiWang, HaoyuKawakami, HiroshiWang, HualinKawakami, SusumuWang, Jeh-jengKawasaki, KiyonoriWang, JianhuaKawczak, PiotrWang, Jing-QuanKazuhito, SatomuraWang, KunKean, James DWang, SaiKebriaei, RaziehWang, Sheng-yiKędziora, AnnaWang, Shu-YinKelarakis, AntoniosWang, SongboKellogg, Joshua J.Wang, TaoKelly, DervlaWang, WilliamKenna, Dervla T.D.Wang, XiaohongKerekes, GyorgyWang, XiuminKeyzers, RobWang, XuKhan, Mohd MohsinWang, ZhejunKhanal, RajendraWang, ZhijunKhattab, MuhammadWang, ZhiqiangKhrustalev, VladislavWang, ZhuoKhurshid, ChrowWareth, GamalKieffer, NicolasWasyl, DariuszKierończyk, BartoszWdowiak-Wróbel, SylwiaKikuchi, HaruhisaWegrzyn, AlicjaKikusato, MotoiWegrzyn, GrzegorzKilonzo-Nthenge, AgnesWei, DongqingKim, Chu-YoungWei, HongpingKim, ChyerWelling, MickKim, DoyoonWełnicki, MarcinKim, Han-NaWertz, Philip W.Kim, Hwa-jungWessely-Szponder, JoannaKim, Jae-gyuWeyand, NathanKim, Jae-SeokWeynberg, KarenKim, Ki-YoungWhite, Raymond P.Kim, SoochongWhitlock, ChristineKim, Tae KonWhitten, SteveKimura, TadashiWianowska, DorotaKing, MariaWieczorek, PiotrKinoshita, AkiyoshiWiesner, JacquelineKirchberger, PaulWijk, Rob Christiaan VanKitano, YoshikazuWilkinson, JennyKitao, TomoeWillett, JuliaKiu, RaymondWilliams, LisaKlewicka, ElzbietaWilson, PeterKlyczek, KarenWinders, HanaKnowles, GraemeWindshügel, BjörnKo, Kwan SooWink, JoachimKo, Wen-ChienWinkler, HeinzKobayashi, NobumichiWitte, WolfgangKobayashi, ScottWitulski, BernhardKobuchi, ShinjiWłodarczyk, MaciejKöck, RobinWnuk, MaciejKocsis, BelaWójciak, MagdalenaKohlhof, HendrikWojtkowska, MałgorzataKoirala, RanjanWojtyczka, RobertKokkinakis, Emmanouil N.Wolny-Koładka, KatarzynaKolar, MitjaWong, ChungKoliarakis, IoannisWróbel, AgnieszkaKomaki, HisayukiWróbel, TomaszKondru, NaveenWróblewska, AgnieszkaKonstantinidis, Theocharis G.Wu, JulieKonto-Ghiorghi, YoanWu, KailiKontsevaya, IrinaWu, Yu-WeiKordalewska, MilenaWujec, MonikaKorneev, DenisXie, HaiboKosik-Bogacka, DanutaXing, FuguoKostoulias, XeniaXiong, JiaKotlowski, RomanXu, JianKotzamanidis, CharalambosXu, JinKovacs, RenatoXu, ZhenboKowalczuk-Vasilev, EdytaXuan, Tran DangKox, MatthijsXue, TingKoyama, ToshihiroYagüe, PaulaKoyama, YuYajima, ShunsukeKozačinski, LidijaYamaki, JasonKozłowska, MariolaYan, DayunKramer, TobiasYan, XiaohuiKrezel, AndrzejYanagisawa, NaokoKrishna, GokulYang, Angela Wei HongKrishnakumar, DevadasYang, ChunhuaKrishnan, NatrajYang, InhoKröger, CarstenYang, KunKrólicka, AleksandraYang, LeiKrutova, MarcelaYang, Shih ChunKrystyna, Skalicka - WoźniakYang, XuKrzych, LukaszYannick, TauranKrzyściak, WirginiaYaoita, YasunoriKrzyżek, PawełYaraki, Mohammad TavakkoliKuczyńska-Wiśnik, DorotaYasa, OncayKuhnert, EricYero, DanielKujan, OmarYeung, Andy Wai KanKulik, Eva M.Yin, ZhiminKulkarni, KetavYoganathan, SabesanKumar, AnandYokomichi, HiroshiKumar, RajYokota, Shin-ichiKumar, SandeepYong, Kar WeyKumar, SujeetYoshida, AkihiroKumar, SunilYu, BingranKuźnik, NikodemYu, LuKwiatkowski, PawelYuan, QingbinLa Clair, JamesYuan, YuanLa Penna, GiovanniYue, MinLa Rosa, RuggeroYukl, Erik T.La Teana, AnnaYun, Hwi-YeolLaabei, MaisemYun, JonghyunLabrou, NikolaosZacharis, Constantinos K.Lagatolla, CristinaZaffina, SalvatoreLaGrow, Austin L.Zaini, PauloLai, Pin-KuangZampieri, MattiaLamas, AlexandreZanetti, StefaniaLambregts, Merel M. C.Zaragoza, WilliamLamers, ChristinaZarb, PeterLaneri, SoniaŻarowska, BarbaraLangford, BradleyZarrelli, ArmandoLaranjo, MartaZarrilli, RaffaeleLaskowska, EwaZarrintaj, PayamLaudadio, EmilianoZavattaro, ElisaLe Brun, Anton P.Zawadzka, KatarzynaLe Gall, TonyZecconi, AlfonsoLe Guern, RemiZechiedrich, LynnLeache, LeireZeineldin, MohamedLeal, FernandoZeleny, ReinhardLedesma, Ana EstelaZeng, JILee, Brian R.Żesławska, EwaLee, Charles CCZha, GaofengLee, Ching-ChiZhang, ErshuaiLee, HyunZhang, FanLee, Jeong SangZhang, HuaweiLee, Jong BongZhang, JileiLee, Tzu-TaiZhang, JinLee, Wan-KyuZhang, JinyangLei, BenfangZhang, TingLeitão, Jorge H.Zhang, TingtingLelešius, RaimundasZhang, XiaoyuLemay, GuyZhang, YoumingLemée, LaurentZhang, YueLemercier, GillesZheng, ShilongLenhard, Justin R.Zhong, CuiqingLentini, GiovanniZhou, BangjunLeonzio, GraziaZhou, MiLetek, MichalZhou, Wen-WenLi, AndongZhu, JingenLi, BingZhu, LuchangLi, GangZhuangqiang, GaoLi, Hou-JinZielińska-Pisklak, MonikaLi, Wen-WuZimmerman, TahlLi, WenyiZiółkowski, HubertLi, XianjiangŻołek, TeresaLi, Xian-ZhiZowalaty, Mohamed E. ElLibralato, GiovanniZuk, MagdalenaLiebchen, Uwe


